# The 19 kDa *Mycobacterium tuberculosis* Lipoprotein (LpqH) Induces Macrophage Apoptosis through Extrinsic and Intrinsic Pathways: A Role for the Mitochondrial Apoptosis-Inducing Factor

**DOI:** 10.1155/2012/950503

**Published:** 2012-12-19

**Authors:** Alejandro Sánchez, Patricia Espinosa, Teresa García, Raúl Mancilla

**Affiliations:** Departamento de Inmunología, Instituto de Investigaciones Biomédicas, Universidad Nacional Autónoma de México, Circuito Escolar S/N, Ciudad Universitaria, 04510 México, DF, Mexico

## Abstract

We describe the association of caspase-dependent and caspase-independent mechanisms in macrophage apoptosis induced by LpqH, a 19 kDa *Mycobacterium tuberculosis* lipoprotein. LpqH triggered TLR2 activation, with upregulation of death receptors and ligands, which was followed by a death receptor signaling cascade with activation of initiator caspase 8 and executioner caspase 3. In this caspase-mediated phase, mitochondrial factors were involved in loss of mitochondrial transmembrane potential (ΔΨm), release of cytochrome c, and caspase 9 activation. Interestingly, a caspase-independent pathway was also identified; by immunoblot, the mitochondrial apoptosis inducing factor (AIF) was demonstrated in nuclei and cytosol of LpqH-treated macrophages. Confocal microscopy revealed translocation of AIF to the nuclei of the majority of apoptotic cells. These findings emphasize the complex and redundant nature of the macrophage death response to mycobacteria.

## 1. Introduction

Macrophage (MO) apoptosis during *Mycobacterium tuberculosis *(Mtb) infection has focused much attention on its possible role in disease pathogenesis. A number of findings support this view; it has been shown that virulent mycobacterial strains are less apoptogenic than their attenuated counterparts [1] and MO proapoptotic genes are downregulated by virulent strains while the opposite occurs with avirulent mycobacteria [2]. In contrast with latent infection, in active TB patients the expression of genes that promote apoptosis is diminished [3]. These data and the identification of genes that inhibit MO apoptosis in some Mtb mutants [4] have added further support to the view that the ability of Mtb to prevent the apoptotic death of host cells is a virulence trait aimed to preserve its cellular niche. On the other hand, that MO apoptosis represents an innate immune response of the host is suggested by decreased viability of bacilli in apoptotic MO [5–7]. There is also evidence that dendritic cells that have ingested apoptotic MO infected by Mtb can activate T cells through a process known as cross-priming which results in the activation of CD8+ T cells [7, 8]. It is also of interest the identification of Mtb proapoptotic mutants that induce higher T cell immunity which favors the control of infection [9].

Knowledge about the mechanisms involved in the death of mycobacteria-infected MOs has greatly increased during the last decade. Initially, it was characterized as extrinsic type, caspase-dependent apoptosis with TNF-*α* activation [10, 11]. More recent studies have come to show that MO apoptosis in mycobacterial infection is a complex and variegated process. Some reports document the participation of the intrinsic or mitochondrial pathway. Infection with attenuated Mtb strains results in mitochondrial outer membrane permeability with release of cytochrome c and activation of caspase 9 [12, 13]. Recently, the endoplasmic reticulum stress and lysosome pathways have been implicated in macrophage apoptosis provoked by mycobacteria [14]. It has been also reported that host cell death might show features of necrosis particularly with increased bacillary loads [15]. These observations could suggest that mycobacteria instead of apoptosis favor necrosis as a mechanism of dissemination and survival.

A few mycobacterial molecules involved in macrophage apoptosis have been identified; among these are LpqH [16, 17], ESAT 6 [11], PE_PGRS33 [18], and PstS-1 [19]. We undertook this study with the aim of knowing better the biochemical pathways used by LpqH to induce MO apoptosis, specially to know if mitochondrial factors were involved. LpqH is interesting for several reasons; it is one of the few mycobacterial proteins, which in addition to acyl groups possess mannose residues [20]. Recently, we demonstrated that LpqH behaves as an adhesin that interacts with the mannose receptor to promote phagocytosis of mycobacteria [21]. LpqH induces T cell-mediated immunity, although it might also behave as a TLR2 agonist that downregulates antigen presentation to T cells [22].

From the above data, it is clear that the death of mycobacteria-infected MOs is a relevant, mechanistically complex phenomenon. To this complexity contribute findings we present herein. We show that in addition to TLR2 dependent, death receptor-mediated apoptosis, LpqH triggers an intrinsic or mitochondrial pathway, with the participation of cytochrome c and the apoptosis-inducing mitochondrial factor (AIF), a previously unrecognized mechanism of MO cell death induced by Mtb.

## 2. Materials and Methods

### 2.1. Materials

Murine monoclonal antibodies (mAbs) to human TNF-*α* (clone 28401) and human TNFR1 and human TNFR2 (clone 22221) were purchased from R&D Systems (Minneapolis, MN, USA); mAbs to human Fas (clone ZB4) and FasL (clone B-R17), caspase 8, caspase 9, and caspase 3 were purchased from Upstate Cell Signaling (Lake Placid, NY, USA); mAb to human TLR2 (clone TL2.1) were from eBioscience (San Diego, CA, USA) and TLR4 (clone HTA-125) was obtained from Santa Cruz Biotechnology (Santa Cruz, CA, USA). A mouse monoclonal antibody to the human-apoptosis inducing factor (AIF) was obtained from Santa Cruz Biotechnology (clone E20). A goat polyclonal antibody to human cytochrome c was purchased from Santa Cruz Biotechnology (clone C-20). Horseradish peroxidase-conjugated control isotype antibodies to goat IgG and to mouse IgG were from Dako (Carpinteria, CA, USA). A cell-death detection enzyme-linked immunosorbent assay (ELISA) Plus was obtained from Roche Diagnostics (Penzberg, Germany). A specific cell-permeable pancaspase inhibitor Z-VAD-FMK was obtained from BD Pharmingen (San Diego, CA, USA). Ficoll-Hypaque was from Amersham Biosciences (Piscataway, NJ, USA); polymyxin B, Ponceau red, and dimethyl sulfoxide (DMSO) were from Sigma-Aldrich (St. Louis, MO, USA). A subcellular protein fractionation kit fractionation was obtained from Thermo Scientific (Rockford, IL, USA). The fluorescent lipophilic dye 3,3′ dihexyloxacarbocyanine iodide (DiOC6) was purchased from Molecular Probes (Eugene, OR, USA).

### 2.2. Mycobacteria and Isolation of LpqH

A native *Mycobacterium smegmatis *strain (mc2155) and its counterpart transformed by electroporation with the plasmid p16R1 containing a 1.8 kb SmaI fragment that includes the structural gene encoding the LpqH lipoprotein were kindly donated by Y. Zhang (MRC Tuberculosis and Related Infections Unit, Hammersmith Hospital, London, UK). *M. smegmatis *strains were grown for 4-5 days in Middlebrook 7H9 medium (BBL, Becton-Dickinson, Cockeysville, MD, USA) supplemented with 2% glucose and hygromycin B (50 mg/mL). Bacteria were harvested by centrifugation and washed with ice-cold sodium phosphate buffer (10 mM/L). To isolate LpqH, cell-wall fractions were obtained by sonication of bacteria at 20 KHz in iced water (five cycles of 5 min each). Proteins were resolved by 12% or 15% sodium dodecyl sulfate polyacrylamide gel electrophoresis (SDS-PAGE) and transferred to nitrocellulose. The 19 kDa LpqH band was identified by immunoblot with mAb IT-19, which was donated by TB Vaccines Testing and Research Materials Contract, Colorado State University. In order to obtain LpqH to induce apoptosis, cell wall fractions were obtained by sonication and 30 *μ*g protein was mixed with an equal volume of reducing sample buffer (0.05 mM EDTA, 0.1% SDS, 1% glycerol, 10% 2-mercaptoethanol, and 0.5 mM/mL Tris-HCl pH 6.8), heated for 5 min at 95°C, and loaded into 1 *μ*L 1.5 mm slot 20 cm long gels. After electrophoresis, proteins were transferred to nitrocellulose sheets and stained with Ponceau red to identify the 19 kDa band; the identity of this band was confirmed in parallel blots with the IT-19 mAb. Thereafter, the band was excised, solubilized in DMSO, and precipitated with an equal volume of carbonate/bicarbonate sodium buffer (0.05 M, pH 9.6). The pellet was rinsed three times with ice-cold PBS and aliquots were stored at 20°C. Protein concentration was determined with the Lowry method using BSA as standard. Native *M. smegmatis *cell wall fractions were electron-transferred to nitrocellulose membranes and a 19-kDa strip was excised and processed as above to be used in apoptosis assays as a control. Western blots with the IT-19 mAb failed to demonstrate LpqH in cell walls of native *M. smegmatis *bacilli (not shown).

### 2.3. Apoptosis Assays to Determine Capacity of LpqH to Kill Human Monocyte-Derived Macrophages

Blood samples were obtained from healthy donors and mononuclear cells were separated by the Ficoll-Hypaque gradient centrifugation. Cells were cultured in Petri dishes (Corning Inc., Corning, NY, USA), in RPMI-1640 supplemented with 20% fetal bovine serum, 5 mM/L L-glutamine, and 5 *μ*g/mL penicillin-streptomycin, at 37°C in a humidified 5% CO_2_ atmosphere. After overnight incubation at 37°C, adherent cells were cultured with 10% fetal bovine serum. This step is considered necessary to get rid of nonadherent cells, death cells, and nonmacrophagic cells. At 7 days of culture and after treatment with RPMI-1640 containing EDTA (5 mM/L), at 4°C for 10 min, activated macrophages were gently detached with a cell scraper. After rinsing, cells (5 × 10^5^) were placed in 12-well flat-bottom plates (Corning Inc., Corning, NY, USA); at this time, trypan blue exclusion showed >95% cell viability. Thereafter, cells were incubated with 0.5, 5, or 50 *μ*g of LpqH for 1 or 24 h at 37°C. For control, cells were incubated with 100 *μ*g protein present in 19 kDa region of native *M. smegmatis *cell wall. Apoptosis was measured by ELISA with a cell-death detection kit according to the manufacturer's instructions. Plates were analyzed with an ELISA reader (Bio-Tek Instruments, Winooski, VT, USA).

### 2.4. Detection of Caspase Activation by Immunoblot and Inhibition Assays with a Pancaspase Inhibitor

Macrophages (2 × 10^6^) were incubated with 5 *μ*g LpqH for 1 h. Thereafter, whole cell extracts were obtained after cells were lysed and vortexed with 100 *μ*L radioimmunoprecipitation assay buffer (50 mM Tris-HCl, pH 7.0, 50 mM NaCl, 1% NP40, 0.5% sodium deoxycholate, and 0.1% SDS) and let stand for 20 min at 4°C. Cell lysates were centrifuged (34025.6 g for 15 min at 4°C) and the supernatant was obtained; 35 *μ*g protein were resolved by 17.5% SDS-PAGE and transferred to polyvinylidene difluoride (PVDF) membranes; after blocking with 5% nonfat milk in PBS, membranes were incubated with mAbs to caspase 8, 9, and 3 (dilution 1/1000), overnight, at 4°C. After rinsing, membranes were incubated with horseradish peroxidase-conjugated secondary antibodies (dilution 1/1000), for 1 h, at room temperature. Reactive bands were visualized by chemiluminescence with SuperSignal West Dura kit (Pierce, Rockford, IL, USA). Inhibition of caspase activity was measured as follows. Monocyte-derived macrophages (1 × 10^6^) were preincubated for 30 min at 37°C with 20 *μ*M of the pancaspase inhibitor Z-VAD-FMK. After rinsing, cells were incubated with 5 *μ*g LpqH for 1 or 24 h at 37°C. Apoptosis was measured by ELISA as described before.

### 2.5. Quantitation of Death Receptors TNFR1, TNFR2, and Fas and Their Ligands TNF-*α* and Fas

To quantitate TNF-*α* production, 5 × 10^5^ cells were incubated for 1 h with 5 *μ*g LpqH or with 100 *μ*g protein present in native *M. smegmatis *19 kDa strips. Supernatants were collected by centrifugation at 390.6 g for 5 min and TNF-*α* was measured after 0, 5, 15, 30, 45, and 60 min treatment, with an ELISA kit according to the manufacturer's instructions (Assay Designs, Inc, Ann Arbor, MI, USA). Duplicate samples were analyzed with an ELISA reader and compared with a standard curve. The expression of death receptors and their ligands was determined by a cell-surface ELISA method. After induction of apoptosis by incubation of cells (5 × 10^5^) with 5 *μ*g LpqH for 1 h at 37°C, cells were rinsed with PBS containing 1% fetal bovine serum and 0.1% NaN_3_. The mouse mAb used were: to human TNFR1 (1 *μ*g/mL, dilution 1/100), human TNFR2 (2 *μ*g/mL, dilution 1/100), human Fas (1 *μ*g/mL, dilution 1 : 100), and human FasL (0.5 *μ*g/mL, dilution 1/100). After apoptosis induction, cells were extensively rinsed and incubated for 1 h with each of the above antibodies. For control, irrelevant isotype antibodies were used. Thereafter, cells were rinsed with PBS and incubated for 1 h with peroxidase-labeled goat or mouse anti-IgG antibodies diluted 1/1000. Finally, 100 *μ*Lof ABTS solution were added to the cells. Optical densities at 405 nm was read by an ELISA reader (Asys Expert Plus, HyTech, Austria).

### 2.6. Inhibition Assays to Assess the Role of TLR2 and TLR4 and of Death Receptors and Their Ligands in LpqH-Induced Macrophage Apoptosis

Cells were preincubated for 30 min at 37°C with blocking mAb to human TNF-*α* (1 *μ*g/mL), human TNFR1 (5 *μ*g/mL), or human TNFR2 (1.5 *μ*g/mL). Inhibition assays were also performed with blocking mAbs to human Fas (500 ng/mL) and to human FasL (32 ng/mL). To study the role of Toll-like receptors in the induction of apoptosis, cells were preincubated with blocking mAbs (20 *μ*g/mL) to TLR2 and to TLR4. Control isotype antibodies were used. After preincubation with antibodies, without rinsing, 5 *μ*g of LpqH was added to the cells to induce apoptosis which was analyzed by ELISA as above.

### 2.7. Evaluation of Mitochondrial Membrane Potential by Flow Cytometry with DiOC6

In order to analyze the participation of the mitochondria, we analyze the mitochondrial membrane potential by flow cytometry using the cationic lipophilic fluorochrome 3,3′-dihexyloxacarbocyanine iodide (DiOC6). Loss in DiOC6 staining indicates disruption of the mitochondrial inner transmembrane potential (ΔΨm) which is often associated with apoptosis [23]. Cells (1 × 10^6^/mL) were incubated with 5 *μ*g LpqH for 1 and 24 h and washed three times with PBS, and 40 nM DIOC6 was added to pellet for 30 min at 37°C in the dark. DiOC6 was prepared from a 40 mM stock solution in DMSO which was diluted with PBS, pH 7.4, to a 400 nM working solution. Cells were diluted with PBS to a final volume of 1 mL and analyzed by flow cytometry.

### 2.8. Subcellular Fractionation and Immunoblot to Determine Mitochondrial Release of Cytochrome c and AIF

MOs treated with 5 *μ*g LpqH for 1 and 24 h were subjected to a subcellular fractionation procedure. Cytosolic and nuclear proteins were extracted using a subcellular protein fractionation kit, following protocols from the manufacturer (Thermo-Scientific). Protein concentrations were measured with the Bio-Rad DC protein assay (Hercules, CA, USA). Proteins (50 *μ*g) were separated by SDS-PAGE in 7.5% polyacrylamide gels and transferred to nitrocellulose membranes. After blocking in 5% non-fat dried milk at room temperature for 1 h, membranes were incubated overnight with a mouse mAb to human AIF diluted 1/200 in TBS with BSA 5% and 0.01% Tween 20; a secondary anti-mouse IgG peroxidase-labeled antibody was used. Different strips were incubated with a polyclonal goat antibody to human cytochrome c, diluted 1/200 in PBS Tween 20, 0.01%, overnight. A secondary antibody to goat IgG labeled with peroxidase was used diluted 1/1000. The SuperSignal West Femto Maximum Sensitivity Substrate chemiluminescence kit was used to reveal reactive bands.

### 2.9. Immunofluorescence to Detect AIF in Nuclei of Macrophages Treated with LpqH

Translocation of AIF from mitochondria to the nucleus during apoptosis was analyzed by immunofluorescence of untreated cells or cells treated with LpqH for 1 and 24 h. Cells were rinsed with PBS and fixed in a solution of 1% paraformaldehyde in PBS. Cell suspensions were placed by cytospin onto glass slides previously treated with poly-L-lysine. Slides were washed once with PBS and a drop a 0.05% saponin solution in PBS was added for 5 min, rinsed with PBS, and incubated for 1 h with the anti-AIF mAb, diluted 1/200, at room temperature in a humidified chamber; slides were rinsed with PBS and incubated for 45 min with a 1/500 dilution of an anti-mouse IgG antibody labeled with CY5. Slides were mounted with ProLong Gold antifade with DAPI (Molecular Probes). Cells were examined with an Olympus BX51 microscope and with a laser scanning confocal microscope LSM 5 Pascal Zeiss with Software 2.8.

## 3. Statistical Analysis

To compare individual experiments, optical density (OD) of cells that were not exposed to the apoptogenic protein was set to 1. All other OD values within an experiment were divided by the OD of untreated control cells to provide a relative apoptosis value in arbitrary units. Data obtained from independent experiments are presented as the mean ± SE, and the differences between conditions were analyzed using Student's *t*-test and Student's paired *t*-test. Differences at *P* < 0.05 and *P* < 0.01 were considered significant.

## 4. Results

### 4.1. In the Apoptotic Death of Human Macrophages Participate Caspase-Dependent Mechanisms

**Figure 1:**
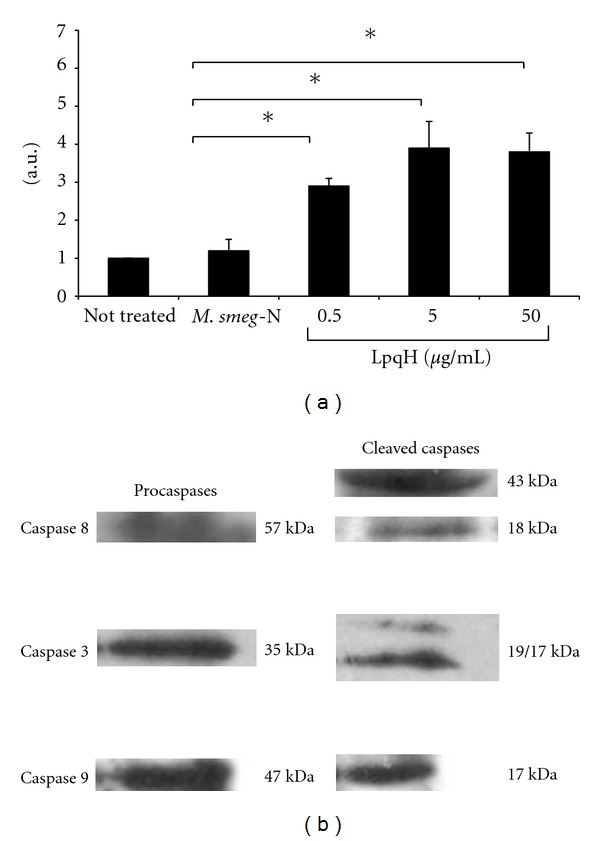
*Mycobacterium tuberculosis *LpqH lipoprotein induces caspase-dependent macrophage apoptosis. (a) Peripheral blood monocyte-derived human macrophages were incubated for 1 h with LpqH. As a control, cells were incubated with 100 *μ*g protein present in 19-kDa nitrocellulose strips of native *M. smegmatis *(*M. smeg-N*), which as seen by immunoblot do not contain LpqH (not shown). Apoptosis was measured with a nucleosome ELISA kit. Apoptosis is expressed as the mean ± SE arbitrary units (a.u.)of four independent experiments. Statistically significant differences are indicated; *P < 0.05. (b) To determine the role of caspases, whole-cell protein extracts of MO made apoptotic by incubation with 5 *μ*g LpqH were separated by SDS-PAGE and immunoblot analysis was performed with antibodies to caspases 8, 3, and 9. Procaspases and cleaved caspases are shown.

Studies in our laboratory with whole bacilli or cell walls showed that a transformed *M. smegmatis *strain that overexpresses the LpqH lipoprotein induces high levels of apoptosis in the human monocytic THP-1 cell line (data not shown). To investigate whether the transfected LpqH was responsible, we incubated human MO obtained from peripheral blood from healthy individuals with 0.5, 5, or 50 *μ*g isolated LpqH. As a control, MOs were incubated with 100 *μ*g protein present in 19-kDa nitrocellulose strips of native *M. smegmatis *which does not contain LpqH as shown by immunoblot (not shown). A nucleosome ELISA method revealed that LpqH induces apoptosis at high rates, significantly higher than those induced by native *M. smegmatis *proteins (Figure 1(a); *P* < 0.05). Assays using polymyxin B ruled out LPS participation (data not shown). To investigate the caspase dependence of apoptosis, protein extracts from apoptotic cells were separated by SDS-PAGE, transferred to PVDF sheets, and incubated with mAb to detect activated forms of caspases. Results revealed procaspases as well as bands corresponding to the activated isoforms of caspases 8, 3, and 9 (Figure 1(b)).

### 4.2. *Mycobacterium tuberculosis *LpqH Activates a Proapoptotic Role in TLR2

**Figure 2:**
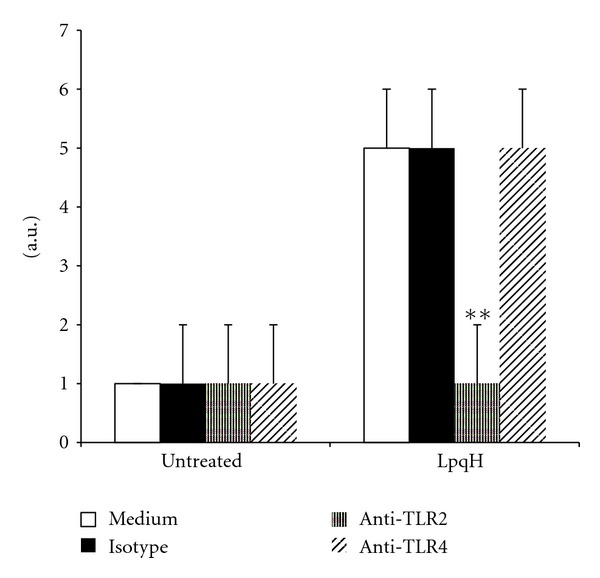
LpqH triggers a TLR2 cell death program. To determine a possible role of TLR2 in MO apoptosis, inhibition assays were performed using a specific mAb known by its ability to block TLR2 activation; 0.5 × 10^6^ cells were preincubated with 20 *μ*g antibody for 1 h and then with 5 *μ*g LpqH to induce apoptosis. A similar procedure was used to analyze the participation of TLR4. Apoptosis was analyzed with a nucleosome ELISA kit. Values are given as the mean ± SE of four independent experiments. Statistically significant differences are indicated; **P < 0.01.

It is well established that TLR2 and TLR4 may behave as cell death receptors and that activation of TLR2 by bacterial lipoproteins may induce apoptosis [24, 25]. To investigate if this occurred in LpqH-treated MO, we carried out inhibition assays with blocking antibodies to these receptors. In cells preincubated with a mAb to human TLR2 and then exposed to 5 *μ*g LpqH, there was a significant reduction in apoptosis levels (Figure 2; *P* < 0.01). This finding indicates that apoptosis is induced by the interaction of TLR2 with motifs present in the mycobacterial lipoprotein. Apoptosis of cells that were preincubated with a mAb to human TLR4 was not modified, a finding in keeping with the fact that this receptor is activated preferentially by LPS motifs and not by lipoproteins [25]. These findings suggest that TLR2 activation acts upstream in the signaling cascade leading to LpqH-induced MO apoptotic death.

### 4.3. Fas and FasL Participate in Macrophage Apoptosis Triggered by LpqH

**Figure 3:**
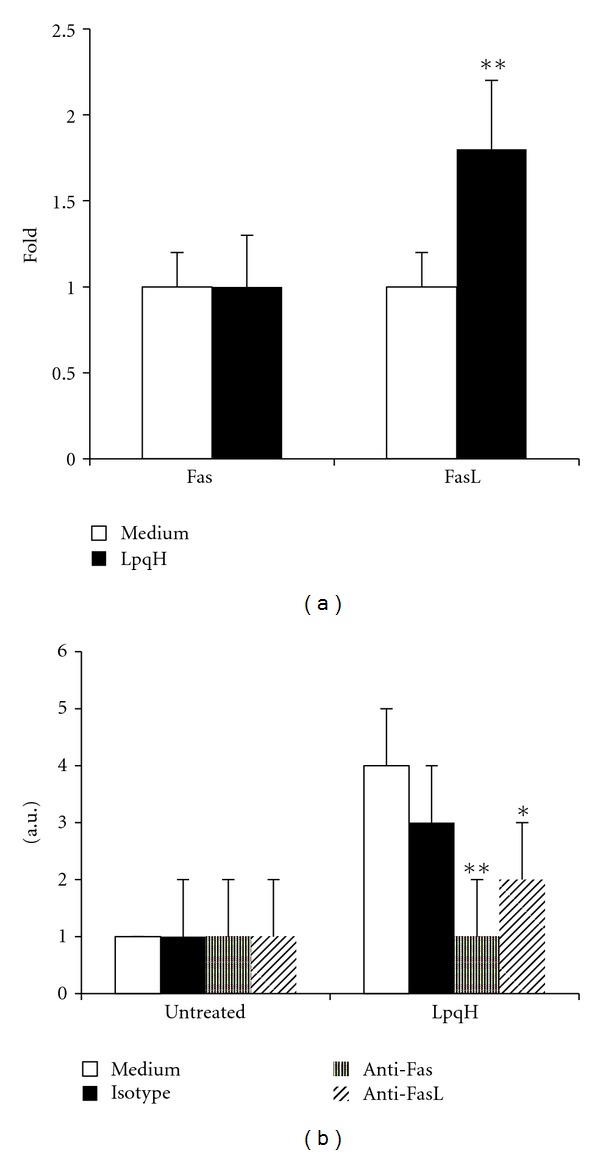
In the LpqH-induced apoptosis participate Fas/FasL. (a) MOs were incubated for 1 h with 5 *μ*g LpqH and the expression of Fas and FasL was quantitated by a cell surface ELISA method using specific mAb. The relative fold increase over untreated cells (set to 1) is shown as the mean ± SE of four independent experiments; statistically significant differences are indicated; **P < 0.01. (b) Inhibition assays to establish the role of Fas and FasL were carried out. MOs were preincubated with neutralizing mAb to Fas and FasL. Thereafter, without rinsing, cells were incubated with 5 *μ*g LpqH for 1 h. The mean ± SE apoptosis level of 3 independent experiments is indicated. *P < 0.05; **P < 0.01.

Information on the role of FasL in macrophage apoptosis induced by mycobacteria is scanty [5]; therefore, we examined the role of FasL and its receptor in LpqH-induced apoptosis. A cell surface ELISA analysis showed that in cells incubated with LpqH, Fas expression was similar to that of untreated cells. In contrast, membrane-bound FasL was significantly increased (*P* < 0.01; Figure 3(a)). This finding is of note because usually FasL is released efficiently from the cell-surface by metalloproteinases [26]. To know if Fas or FasL had a role in apoptosis, inhibition assays were carried out. When MOs were preincubated with blocking mAb against Fas and FasL, the apoptosis levels were significantly decreased (Figure 3(b); *P* < 0.01; *P* < 0.05).

### 4.4. Involvement of TNF-*α* and Its Receptors in Macrophage Apoptosis

**Figure 4:**
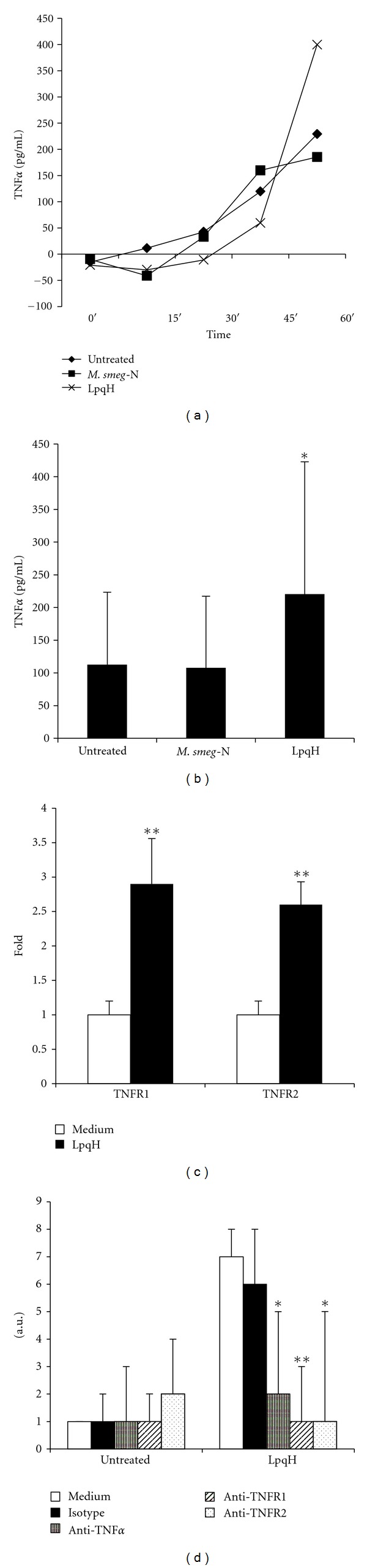
TNF-*α* and their receptors are upregulated in LpqH-promoted apoptosis. (a) MOs were incubated with 5 *μ*g LpqH to induce apoptosis. Control cells were incubated with 100 *μ*g native *M. smegmatis *protein (*M. smeg-N*). At indicated times the culture medium was collected and TNF-*α* production was quantitated by ELISA. (b) Culture media of apoptotic cells and control cells were collected at 60 min and TNF-*α* was measured by ELISA. Values are presented as the mean ± SE of three independent experiments; *P < 0.05. (c) After 1 h incubation of cells with 5 *μ*g LpqH, the expression of TNFR1 and TNFR2 was measured by cell surface ELISA. The relative fold increase compared with untreated cells (here set to 1) is shown as the mean ± SE of three independent experiments; **P < 0.01. (d) MOs were preincubated with neutralizing mAb to human TNF-*α*, TNFR1, and TNFR2. Thereafter, without rinsing, 5 *μ*g LpqH was added to cells for 1 h; apoptosis was measured as described in Materials and Methods. Results are given as the mean ± SE of three independent experiments. Statistically significant differences are indicated; *P < 0.05; **P < 0.01.

TNF-*α* production during LpqH-induced MO apoptosis was measured by an ELISA method in the cultured medium of cells incubated with 5 *μ*g LpqH for 15, 30, 45, and 60 min. Increased time-dependent TNF-*α* values were observed, which were maximal at 60 min (Figure 4(a)). At this time, cells treated with LpqH released TNF-*α* in amounts significantly higher than those of cells incubated with 100 *μ*g native *M. smegmatis *proteins present in the 19-kDa region (Figure 4(b); *P* < 0.05). In view of the importance of TNFR1 and TNFR2 in the cytotoxic capacity of TNF-*α* [27], their expression was analyzed by a cell surface ELISA procedure. Both receptors were overexpressed after treatment with 5 *μ*g LpqH (Figure 4(c); *P* < 0.01). To know whether TNF-*α* and death receptors upregulation was related to the induction of apoptosis, inhibition assays with blocking antibodies were carried out. It was observed that mAbs to TNF-*α* (*P* < 0.05), TNFR1 (*P* < 0.01), and TNFR2 (*P* < 0.01) decreased apoptosis significantly (Figure 4(d)). The latter observation is interesting because TNFR2 is a receptor devoid of cell-death domains [27, 28]. By cell surface ELISA no differences were found in TNF-*α* membrane expression between LpqH-treated and untreated cells (not shown). No effect was observed with an isotype control.

### 4.5. Participation of Mitochondrial Factors in LpqH-Induced Apoptosis

**Figure 5:**
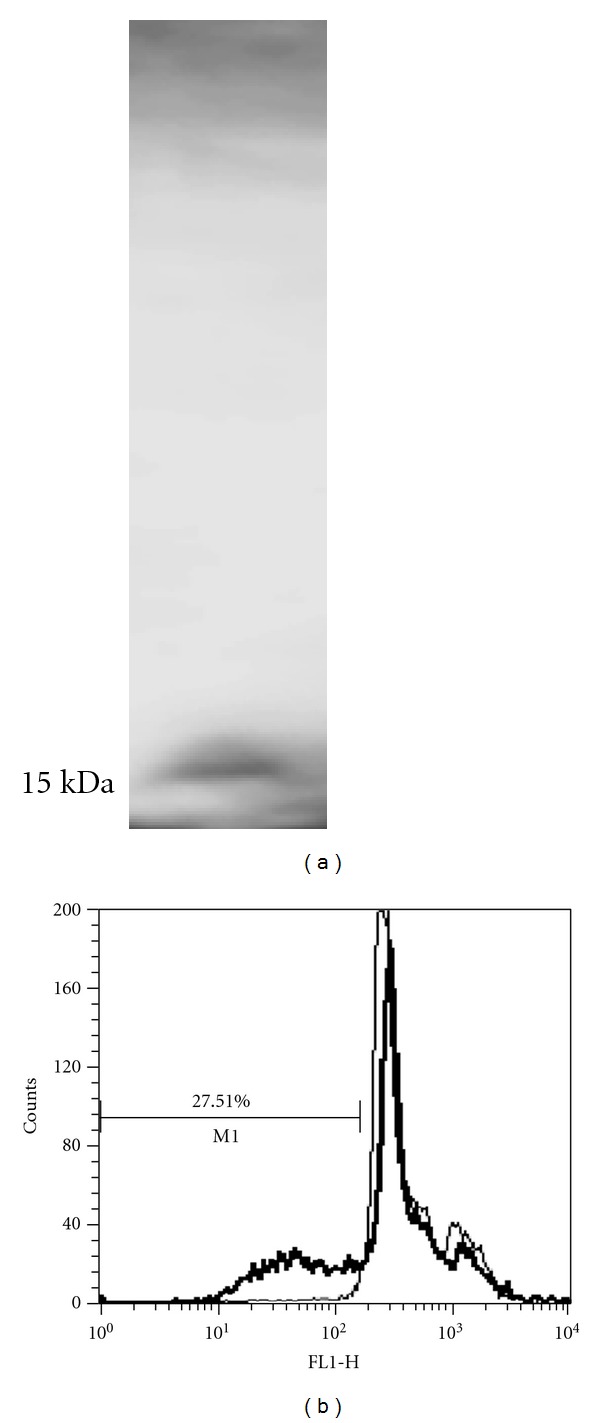
Engagement of mitochondrial factors in the caspase-dependent phase of the death cascade triggered by LpqH. (a) To know if LpqH induces transfer of cytochrome c from the mitochondria to the cytosol, we perform immunoblot of cytosolic fractions with an antibody to human cytochrome c after 24 h LpqH apoptosis. (b) To determine the mitochondrial membrane damage, we analyzed the mitochondrial membrane potential (ΔΨm) by flow cytometry with DiOC6 in cells induced to apoptosis for 24 h; 27.51% loss in DiOC6 staining is observed.

Immunoblot analysis of cells driven apoptotic by LpqH exposure showed activation of caspase 9 (Figure 1(b)) which requires the participation of mitochondrial factors [29]. Activation of caspase 9 requires apoptosome formation which is promoted by cytochrome c when it is released from the mitochondria [29]. Hence, we obtained subcellular fractions from MO treated with LpqH for 24 h to perform immunoblot analysis. In the cytosol, a mAb antibody specific for human cytochrome c revealed the expected presence of a 15 kDa band (Figure 5(a)). To further asses the role of mitochondria, we tested whether LpqH caused a loss in mitochondrial transmembrane potential (ΔΨm) using the cationic lipophilic fluorochrome DiOC6 [30, 31]. In this study, fluorescence microscopy of labeled MO displayed the punctate cytoplasmic pattern characteristic of mitochondria labeling, with no discernible plasma membrane labeling (data not shown). In comparison with control cells, in LpqH-treated cells, there was a 27.51% decrease in DiOC6 labeling (Figure 5(b)), indicating disruption of the mitochondrial inner transmembrane potential (ΔΨm), a change often associated with apoptosis [31].

### 4.6. Caspase-Independent Mechanisms Are Involved in LpqH-Induced Apoptosis: A Role for the Apoptosis-Inducing Factor (AIF)

**Figure 6:**
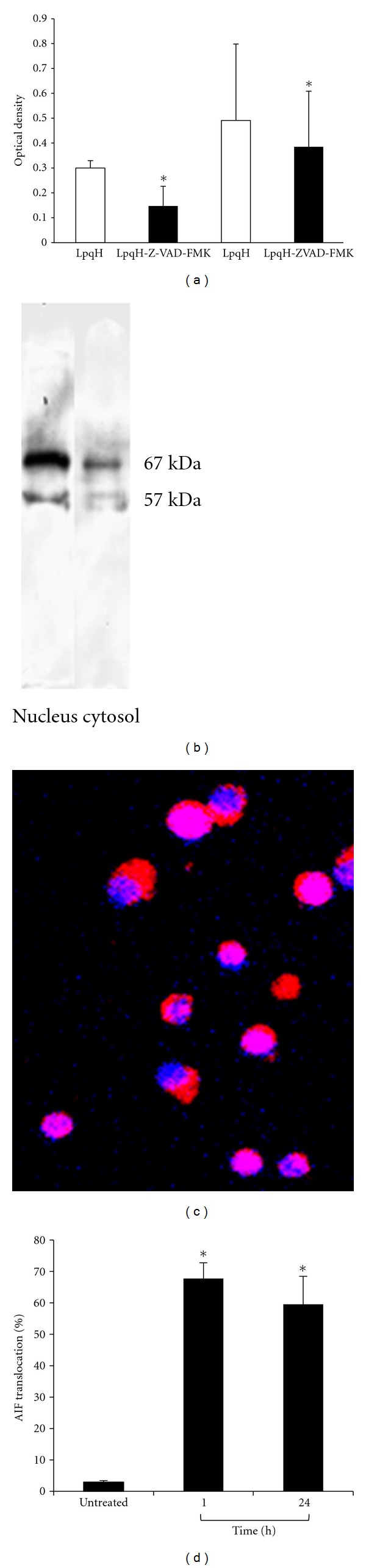
Caspase-independent mechanisms with AIF relocation are involved in LpqH-induced MO. (a) MOs were preincubated with the pancasphae inhibitor Z-VAD-FMK for 30 min and thereafter apoptosis was induced with 5 *μ*g LpqH for 1 and 24 h; apoptosis was measured by a nucleosome ELISA kit. At 1 h apoptosis (left) inhibition was of 49.08% and at 24 h of 19.41% (right). Data from four independent experiments are expressed as mean ± SE of the optical density found; *P < 0.05. (b) Cells, untreated or treated with LpqH for 24 h, were subjected to subcellular fractionation and immunoblot with a mAb to human AIF using cytosolic and nuclear fractions. (c) For immunofluorescence, after 1 and 24 h apoptosis induction, MOs were incubated with a mAb to human AIF labeled with CY5 (red fluorescence); nuclei were labeled with DAPI (blue fluorescence). By confocal microscopy, photographs were taken at the midsection of at least 100 cells per condition; photographs were analyzed and merged using the National Institutes of Health ImageJ software (Bethesda, MD, USA). AIF is seen in nuclei as magenta fluorescence frequently massive resulting from the colocalization of CY5 and DAPI; ×40, original magnification. (d) Epifluorescence was used to estimate the percentage of cells showing AIF nuclear translocation at 1 and 24 h; at least 500 cells randomly chosen were photographed and analyzed with ImageJ; *P < 0.05.

In view of the implication of mitochondrial factors in the induction of caspase-dependent apoptosis, we considered possible that caspase-independent factors could be also involved in LpqH-induced MO death. Therefore, we carried out assays with the pancaspase inhibitor Z-VAD-FMK (Figure 6(a)). In cells treated with LpqH for 1 h, there was 49.08% reduction in apoptosis (*P* < 0.05), while in cells treated with LpqH for 24 h, apoptosis reduction was of only 19.41% (*P* < 0.05). This suggested a role for a caspase-independent mechanism. It is well established that after a variety of apoptotic stimuli, AIF is rapidly released from the mitochondria and relocated to the nuclei to induce chromatin condensation and cell death which is unresponsive to caspase inhibitors [32, 33]. Therefore, we investigated by immunoblot the subcellular distribution of AIF in cells treated with LpqH for 24 hr. In nuclei and cytosol, a doublet band was observed, one of them of 57 kDa, a molecular weight that corresponds to activated or truncated AIF (Figure 6(b)); the upper band corresponds to uncleaved AIF [32, 33]. To confirm these findings, we performed immunofluorescence with a CY5-labeled mAb to AIF; many confocal microscopy photographs taken at the central part of the cell were examined. In cells treated with LpqH for 1 and 24 h, translocation of AIF was seen in many nuclei as magenta fluorescent spots or massive fluorescence (Figure 6(c)); magenta fluorescence results from the colocalization of CY5 (red fluorescence) which labels AIF and DAPI nuclear staining (blue fluorescence). Unaffected or not treated control cells showed AIF emitting CY5 red fluorescence, localized to the cytoplasm, with a punctate pattern characteristic of mitochondrial labeling (Figure 6(c)). Epifluorescence was used to quantitate AIF nuclear translocation in 3 independent experiments; photographs were taken in several randomly chosen fields, and for quantitative analysis at least 500 cells per condition were scored as showing or not AIF in nuclei (Figure 6(d)); similar percentages of cells were positive for AIF at 1 (67.7%) and 24 h (59.5%).

## 5. Discussion

Although the apoptogenic capacity of LpqH has been reported before [16, 17], this study gives new insight about the mechanisms involved. It is shown that both extrinsic and intrinsic mechanisms, including AIF relocation, act together to provoke the apoptotic death of human MO challenged with LpqH. In the extrinsic or receptor-mediated pathway, a novel observation was the overexpression of membrane-bound FasL, a protein that is cleaved with high efficiency from the cell surface by metalloproteinases [26]. It is interesting that membrane-bound FasL is a considerably more potent inducer of apoptosis than soluble FasL [34]. This property could be relevant in TB since tuberculous granulomas contain numerous macrophages that express surface FasL [35]; this raises the possibility that these FasL armed cells may kill by direct contact Fas-expressing cells within the granuloma. Another new observation was the upregulation not only of TNF-*α* but also of both TNFR1 and TNFR2 receptors; this increased expression was significant as shown by inhibition assays with blocking mAb which reduced apoptosis levels significantly. This finding poses the question of how TNFR2 participates in LpqH induced cell death considering that in contrast to TNFR1, TNFR2 lacks a death domain. It has been reported that TNFR2 may participate in apoptosis by inducing endogenous TNF-*α*,depleting antiapoptotic signals, or increasing TNFR1 expression [27, 28].

In this study we found that in the MO death induced by LpqH, TLR2 signals as an upstream activator of the apoptosis cascade. This was demonstrated in inhibition assays with blocking antibodies. Like other bacterial lipoproteins LpqH is a TLR2 agonist that can either activate cells to produce proinflammatory cytokines [22] or trigger the death of host cells [16, 17]. The motifs in LpqH that active TLR2 are unknown; acyl groups in the lipoprotein are likely candidates, as suggested by studies with artificial bacterial lipoprotein motifs [36]. When TLR2 activated by LpqH will behave as a cell death receptor, as shown in this study, and when it will mediate proinflammatory signals remain as intriguing as yet unanswered questions.

An interesting finding of this study was the apparent cooperation found between extrinsic and intrinsic pathways to provoke MO apoptosis. Indeed, activation of caspase 9 and cytochrome c released from mitochondria into the cytosol were demonstrated. When a given apoptotic stimulus induces outer mitochondrial membrane (OMM) permeability, cytochrome c is released from the mitochondria to the cytosol where it mediates apoptosome formation; this macromolecular complex results from the interaction of APAF-1, ATP, and procaspase 9, which is cleaved into its activated form. In turn, active caspase 9 cleaves and activates procaspase 3, thereby triggering the executioner phase of caspase-dependent apoptosis [29]. In addition, as shown by flow cytometry using the lipophilic fluorochrome DiOC6 a reduction of the mitochondrial transmembrane potential was found; this change indicates permeabilization of the inner membrane, an apoptosis feature less frequent than OMM permeabilization but frequently associated with cytochrome c release [37]. The mechanism interconnecting the extrinsic pathway with the mitochondrial pathway in apoptosis induction requires further study. It has been described that caspase 8 might cleave Bid (BH3-interacting domain death agonist), a proapoptotic Bcl2 (B-cell lymphoma 2) family member; then truncated Bid localizes to the OMM where it promotes Bax (Bcl-2-associated X protein) activation, resulting in release into the cytosol of proapoptotic or antiapoptotic molecules from the mitochondria [38].

The above findings featured the participation of the mitochondria in a caspase-dependent mechanism that trigger MO death after LpqH exposure. Since it is well recognized that mitochondria can also release factors involved in caspase-independent cell death [37], we considered this possible. This was also entertained by the limited effect of a pancaspase inhibitor to avoid apoptosis in LpqH-challenged cells. Among the proapoptotic molecules released with OMM permeability is AIF, which has been shown to be frequently involved in caspase-independent apoptosis [39]. AIF is a mitochondrial flavoprotein with a twofold function, in the mitochondria it participates in oxidative phosphorylation, a life function, and, when out of the mitochondria, it provokes cell death [39]. After an apoptotic stimulus, AIF can be cleaved by calpains or cathepsins yielding a truncated 57-kDa form which, once released in the cytosol, relocates to the nucleus to promote chromatinolysis and programmed cell death [33]. In this study, in cells treated with LpqH, AIF was seen by immunoblot in the nuclei and to a lesser extent in the cytosol. In both locations, two bands were detected, one of 57 kDa, possibly corresponding to truncated AIF. In addition, by epifluorescence and confocal microscopy, translocation of AIF to the nuclei of most cells was seen at 1 and 24 h. As far as the mechanisms involved in AIF release, it has been shown that overexpression of PARP-1 generates PAR polymers which colocalize with the mitochondria where they induce liberation of AIF to the cytosol with subsequent translocation to cell nuclei [40]. Another factor that may connect caspase-dependent and caspase-independent pathways is caspase 3, which might cleave nuclear PARP-1 [41].

AIF has focused much attention not only as a model of cell death, but also for its role in pathological conditions. It participates in the death of cancer cells treated with cytotoxic drugs and in various neuropathological processes [42]. Cell death with the participation of AIF has been reported in infectious processes including viral infections such as HIV, [43], rabies [44], and coronavirus [45]. In some bacterial infections, AIF triggers the death of infected cells, including *Helicobacter pylori *[46], pneumococcus [47], and *Coxiella burnetii *infections [48]. Regarding pathogenic mycobacteria, AIF-induced MO death has been reported to occur in THP-1 monocytes and bovine MO infected in vitro with *M. bovis* [49, 50]. To these observations, it should be added the data herein presented showing that AIF can participate in the death of human MO treated with LpqH, a cell wall, 19 kDa lipoprotein of Mtb.

## 6. Conclusion

Our study documents the high capacity and versatility of a Mtb lipoprotein to assure the death of human monocyte-derived macrophages. It also shows the high complexity of the cell death process and how extrinsic and intrinsic pathways of apoptosis cooperate. The most interesting and previously unreported observation was the participation of the mitochondrial apoptosis factor (AIF) in the macrophage death triggered by a Mtb lipoprotein. It follows to study the molecular mechanisms and the significance of the cooperation between death-receptor and mitochondrial mediated apoptosis.
